# FDA-Approved Pharmacotherapy for Weight Loss Over the Last Decade

**DOI:** 10.7759/cureus.29262

**Published:** 2022-09-17

**Authors:** Zarwa Idrees, Ivan Cancarevic, Li Huang

**Affiliations:** 1 Internal Medicine, Icahn School of Medicine at Mount Sinai/NYC Health+Hospitals Queens, New York City, USA; 2 Endocrinology, Icahn School of Medicine at Mount Sinai/NYC Health+Hospitals Queens, New York City, USA

**Keywords:** anti-obesity, weight reducton, semaglutide, liraglutide or saxenda, naltrexone-bupropion, phentermine-topiramate, orlistat, weight-loss intervention, obesity, obesity treatment

## Abstract

Obesity is a recently defined illness whose diagnosis and treatment continue to be stigmatized. Currently, due to lifestyle changes brought on by technological advancements and the wide availability and affordability of high-calorie foods, millions of people around the world suffer from obesity and/or its sequelae. Finding adequate prevention and treatment options would therefore lead to massive improvements in the duration and quality of life of affected individuals. In this review, we searched the PubMed database for studies exploring the safety and efficacy of the five medications currently approved by the FDA for the treatment of obesity. We included only studies pertaining to adult patients that have been published between 2012 and 2022. We found evidence that all the drugs analyzed such as orlistat, phentermine/topiramate, naltrexone/bupropion, liraglutide, and semaglutide appear to be effective in inducing weight loss, with the suggestion that semaglutide may have superior efficacy. However, a massive obstacle in developing treatment guidelines remains the lack of prolonged studies monitoring the long-term safety and efficacy of obesity medications. Nevertheless, in patients at risk of complications from obesity, the benefits of losing fat mass may outweigh the potential side effects associated with these medications and clinicians should prescribe whichever of the FDA-approved pharmacotherapy they deem most appropriate for the patient’s specific set of circumstances.

## Introduction and background

Obesity was first termed a disease in 1760 by English physician Malcolm Flemyng [1]. While there is a stigma associated with obesity, if it is seen as a disease instead of a social parameter, the perspective of the general population might change. Overweight individuals are those with a body mass index (BMI) greater than or equal to 25, while obesity is defined as a BMI of 30 or higher [2]. Obesity is a global epidemic that is still on the rise, affecting billions of people worldwide [3]. WHO reports that more than 1 billion people worldwide are obese, putting them at increased risk of worse health outcomes. Obesity has been associated with diabetes mellitus, cardiovascular disease, hypertension, stroke, decreased female fertility, mental health issues, worse outcomes of COVID-19, increased risk of cancer, and ultimately increased mortality [4-6].

The advancements in technology, a more sedentary lifestyle, along with increased access to high-calorie foods, have become the fundamental cause of the increased prevalence of obesity [7]. Genetic factors also play an important role. Thus, the approach toward obesity is also multifactorial including lifestyle modification, pharmacotherapy, and bariatric surgery. Pharmacotherapy can additionally act as a bridge between lifestyle modification and bariatric surgery.

Obesity and excess weight are preventable and, to an extent, treatable. Clinically significant weight loss is defined as at least a 5% reduction of weight from baseline over a period of three months [8]. Pharmacotherapy should be considered in addition to lifestyle modification in those with a BMI of more than 30 or BMI of 27-30 with at least one weight-related complication (for example Diabetes Mellitus, hypertension, dyslipidemia, coronary artery disease, stroke) according to studies and recommendations by the Endocrine Society [9-10]. Adequate prevention and management could save millions of lives and lead to improved quality of life for a very significant percentage of the global population. The FDA has approved five medications for long-term use for weight loss, namely: orlistat, phentermine-topiramate, naltrexone-bupropion, liraglutide, and semaglutide [10]. We are going to take a closer look at these five medications, including their efficacy and benefits versus risks.

## Review

Methods

This is a narrative literature review. PubMed database was searched using the keywords “pharmacotherapy”, “weight loss”, “FDA-approved”, “orlistat”, “phentermine-topiramate”, “naltrexone-bupropion”, “liraglutide”, and “semaglutide”. Observational and interventional studies written in English investigating the outcomes of obesity management with any of the aforementioned drugs that were published between 2012 and 2022 were included. Articles pertaining to pediatric or obstetric patients, review articles, case reports, case series, and articles written in languages other than English were excluded.

FDA-approved medications

There are currently five FDA-approved medications for the management of obesity [10-12]. A sixth drug, lorcaserin, was approved for weight loss but was later withdrawn, after being in clinical use for eight years, due to concerns of increased risk of cancer [11].

Orlistat

Orlistat is a pancreatic lipase and gastric lipase inhibitor that can lead to a decrease in fat absorption by as much as 30% contributing to weight loss [12,13]. Yu et al. found that administration of orlistat in combination with diet or diet and exercise versus diet alone led to a reduction in body mass index, body weight, waist circumference, and levels of total cholesterol and low-density lipoprotein (LDL) cholesterol [14]. Khera et al. found that 44% of the participants taking orlistat achieved 5% weight loss from baseline compared to only 23% of those receiving placebo (Odds ratio (OR) 2.70, 95% credible interval (CrI) 2.34-3.09, surface under the cumulative ranking curve (SUCRA) 0.22) [15]. At the one-year point, excess weight loss with orlistat compared to placebo was 2.6 kg (95% CrI -3.04 to -2.16 kg) [15]. The XENSOR study by Gorgojo-Martinez et al. showed that, over a period of seven months, both orlistat and liraglutide led to a significant reduction in weight (3.3 kg with orlistat vs 7.7 kg with liraglutide), fasting glucose level, systolic blood pressure, LDL cholesterol, and alanine transaminase [16]. Of the participants taking orlistat, 27.4% lost 5% or more of their baseline weight compared to 64.7% with liraglutide [16]. Grabarczyk found that, after 20 weeks of treatment, patients receiving orlistat experienced a 2.1% decrease in weight from baseline, those receiving phentermine-topiramate 4.1%, phentermine alone 3.6%, whereas only 1.6% weight reduction was reported with MOVE! (Veterans Administration (VA)’s weight management program with at least three clinic visits) (P < 0.05] [17].

Orlistat has been shown to be effective for achieving weight loss of 5% or more from baseline. Its beneficial effect on endothelial function also provides cardiovascular benefits [14]. The addition of medications like orlistat, which are FDA-approved and has an established safety profile, is not only helpful for initial weight loss but also its maintenance. It is an oral medication that most commonly causes gastrointestinal adverse effects, including steatorrhea due to impaired fat absorption, which may lead to a deficiency of fat-soluble vitamins and nutrients [18]. Its safety has not been established in the pediatric population under 12 years old and it is contraindicated for pregnant and breastfeeding women [18,19]. Special consideration should be given to patients with thyroid disease, blood coagulation disorders, epilepsy, and HIV, as orlistat interacts with some anti-epileptics, anti-retroviral medications, levothyroxine, and warfarin, requiring close monitoring in these patients [18]. An informed decision should be made based on the patient’s preferences and requirements. 

Phentermine-Topiramate

Phentermine is a sympathomimetic that suppresses appetite by increasing norepinephrine in the central nervous system. Topiramate, a gamma-aminobutyric acid (GABA) receptor agonist, decreases appetite and increases satiety. It is often used for the treatment of epilepsy and migraine headaches [20,21]. The combination of phentermine with topiramate has been approved by FDA for the management of obesity. Khera et al. found significant weight loss associated with phentermine-topiramate in comparison with placebo [15]. A 5% weight loss was reported in 75% of those receiving phentermine-topiramate vs 23% of those receiving placebo (OR 9.22, 95% CrI 6.63-12.85, SUCRA 0.95) vs 44% of the participants taking orlistat (OR 2.70, 95% CrI 2.34-3.09, SUCRA 0.22) [15]. At the one-year point, excess weight loss compared to placebo was 8.8 kg (95% CrI-10.20 to -7.42 kg), while it was 2.6 kg with orlistat compared to placebo (95% CrI -3.04 to -2.16 kg) [15]. In a study by Shi et al., phentermine-topiramate led to a mean difference (MD) of percentage body weight change of -7.97 (95%CI -9.28 to -6.66), while glucagon-like peptide 1 (GLP-1) agonists showed MD of -5.76 (95%CI -6.30 to -5,21) [22]. In the study, phentermine-topiramate was shown to have the lowest likelihood of discontinuation due to side effects [22]. Grabarczyk performed a study with Veteran Health Administration (VHA) and found that after 20 weeks, phentermine-topiramate led to a 4.1% decrease in weight from baseline vs 3.6% for phentermine alone vs 2.1% for orlistat vs 1.6% in those on MOVE! (P < 0.05) [17]. Over 40% of participants taking phentermine-topiramate achieved 5% weight loss over 20 weeks [17]. Shibuya et al. reported average weight loss with any pharmacotherapy to be 3.45% of body weight from baseline; however, participants taking naltrexone-bupropion lost 2.66 ± 5.03% of body weight compared to 3.63 ± 5.7% with phentermine-topiramate (P < 0.0001) [23]. Lei et al. found phentermine-topiramate effective in the reduction of body weight as well as waist circumference, blood pressure, lipid levels, and plasma glucose levels [24]. The dosage affected the efficacy, and a limiting factor was the association of phentermine-topiramate with nervous system side effects [24]. These studies show phentermine-topiramate to be more effective than placebo and other anti-obesity medications but post hoc analysis of the study by Shi et al. established the superiority of semaglutide for weight loss with similar risks of adverse effects [22].

The combination of phentermine-topiramate allows minimal side effects with proven efficacy for weight loss. The side effects that resulted in discontinuation of treatment included blurry vision, headaches, insomnia, paresthesia, irritability, dizziness, anxiety, and depression [25]. This combination is also contraindicated in pregnant women due to teratogenicity. Special attention should be paid when prescribing this medication to the possibility of acute angle-closure glaucoma, teratogenicity in reproductive-age females, and neuropsychiatric symptoms. The studies have been able to establish the efficacy, but data is lacking regarding long-term side effects. In addition to weight loss and a decrease in waist circumference, it has been shown to be associated with a reduction in lipid levels, plasma glucose levels, and blood pressure. The aforementioned studies suggest that the benefits outweigh the risks of adverse effects by causing weight loss and reduction of weight-related complications when used in an appropriate patient population. However, due to the scarcity of information on long-term side effects, a longer prospective observational study would likely help address some of those concerns.

Naltrexone-Bupropion 

The combination of naltrexone, an opioid receptor antagonist, and bupropion, a norepinephrine-dopamine reuptake inhibitor, is used for the management of obesity [26]. Khera et al. found that 23% of participants taking placebo had 5% weight loss compared to 55% of those taking naltrexone-bupropion (OR 3.96, 95% CrI 3.03-5.11, SUCRA 0.60) [27]. At the one-year point, excess weight loss as compared to placebo was 5.0 kg (95% CrI -5.94 to -3.96). Of the five approved medications, according to a recent meta-analysis, naltrexone-bupropion and liraglutide were found to be associated with the highest odds of treatment discontinuation related to adverse events [15]. Shibuya et al. found that participants taking naltrexone-bupropion lost 2.66 ± 5.03% of body weight compared to 3.63 ± 5.7% with phentermine-topiramate (P < 0.0001) making it non-superior to phentermine-topiramate [23]. 

Discontinuation of treatment related to adverse effects including nausea, vomiting, constipation, dry mouth, and dizziness has been reported [27]. Further limitations include the inability to use naltrexone in patients taking opioid analgesics, while bupropion is contraindicated with medications that lower seizure threshold and monoamine oxidase inhibitors. Like other FDA-approved pharmacotherapy, naltrexone-bupropion can be prescribed for the appropriate patient population to benefit from its effects on weight loss and metabolic profile. However, due to its sympathomimetic effects, it should not be used in patients with uncontrolled hypertension. More studies need to be performed to further assess the long-term safety and benefits related to cardiovascular and metabolic health. 

Liraglutide

Liraglutide is a subcutaneous GLP-1 agonist, which is used daily for the management of diabetes mellitus type 2 and obesity/weight management [20]. A systematic review and meta-analysis by Khera et al. found that 23% of participants taking placebo had a weight loss of 5% compared to 63% of those taking liraglutide (OR 5.54, 95% CrI 4.16-7.78, SUCRA 0.83). At the one-year point, excess weight loss compared with placebo was 5.3 kg (95% CrI -6.06 to -4.52 kg). Liraglutide was one of the two drugs associated with the highest odds of discontinuation of treatment related to adverse events [15]. Rubino et al. found that participants taking semaglutide showed a mean weight change of -15.8% compared to -6.4% in those taking liraglutide and -1.9% with pooled placebo (95% CI -12 to -6.8, P < .001). Participants taking semaglutide showed higher odds of achieving weight loss compared with liraglutide: 10% or higher weight loss was achieved in 70.9% of those taking semaglutide vs 25.6% of those taking liraglutide (OR 6.3, 95%CI 3.5 to 11.2, P <.001), 15% or higher in 55.6% vs 12.0% (OR 7.9, 95% CI 4.1 to 15.4, P < .001), 20% or higher in 38.5 % vs 6.0% (OR 8.2, 95%CI 3.5 to 19.1, P < .001). A similar proportion reported gastrointestinal adverse events; 84.1% with semaglutide and 82.7% with liraglutide. Discontinuation of treatment related to adverse events was 13.5% in those taking semaglutide vs 27.6% with liraglutide [28]. A study by Astrup et al. found that in the first year, participants on liraglutide 3.0 mg lost 5.8 kg more weight than those taking placebo (95%CI 3.7 to 8.0) and 3.8 kg more than those taking orlistat (95% CI 1.6-6.0) (P 0.0001). Participants receiving liraglutide 2.4/3.0 mg for two whole years lost 3.0 kg more than those receiving orlistat (95% CI 1.3-4.7, P < 0.001). The liraglutide 2.4/3.0 mg group also experienced improvements in blood lipid levels and blood pressure along with a 52% decreased two-year prevalence of prediabetes and a 59% decreased prevalence of metabolic syndrome [29]. Nausea and vomiting were the most frequently reported side effects [29]. Gorgojo-Martínez et al. found liraglutide to be more effective at weight loss as well as obesity-related metabolic and cardiovascular complications than orlistat [16]. 

Liraglutide is especially beneficial in overweight or obese diabetic patients as it has also been established as an effective therapy for better glycemic control in type 2 diabetics as an adjunct to oral anti-diabetic medications and/or insulin [30]. The pitfall is the route of administration; oral therapy is generally preferred by the patient over subcutaneous therapy but with adequate counseling and education regarding self-administration, these concerns can be alleviated. Once-daily dosing in comparison to weekly dosing of semaglutide also creates comparatively more inconvenience. Discontinuation of treatment has been reported as well due to gastrointestinal side effects, thus making it a favorable option for only those who can tolerate it. Many clinicians are now comfortable prescribing liraglutide in overweight diabetic patients, but they seem to be hesitant to use it for obesity without diabetes. Patients can benefit greatly from the weight loss effects of liraglutide, and its use should be promoted in the appropriate patient population. 

Semaglutide

Semaglutide is a long-acting GLP-1 receptor agonist that is administered subcutaneously once weekly for the management of diabetes mellitus and has been shown to also induce weight loss [31]. Several trials have been conducted to study the effectiveness of this medication to induce weight loss when added to lifestyle modification. The semaglutide treatment effect for people with obesity (STEP) program includes five phase-3 trials to evaluate Semaglutide 2.4 mg once weekly for the degree of weight loss, efficacy, and tolerability [32]. Wadden et al. found that semaglutide was more effective than placebo for weight loss when used as an adjunct to lifestyle modification, the STEP3 randomized clinical trial showed a weight change of -16.0% with semaglutide vs -5.7% with placebo (95%CI -12.0 to -8.6, P < .001) [31]. The STEP4 randomized clinical trial showed weight change with subcutaneous semaglutide was -7.9% as compared to +6.9% with placebo (95%CI -16.0 to -13.5, P < .001) [33]. Improvement was also shown with semaglutide vs placebo in waist circumference -9.7 cm (95% CI -10.9 to -8.5 cm, P < .001), systolic blood pressure -3.9 mmHg (95%CI -5.8 to - 2.0 mmHg, P < .001), and physical functioning score SF-36 2.5 (95% CI 1.6 to 3.3, P < .001). Rubino et al. found semaglutide to be more effective than liraglutide for weight loss, participants taking semaglutide showed a mean weight change of -15.8% as compared to -6.4% in those taking liraglutide (95%CI -12 to -6.8, P < .001) [28]. Gastrointestinal side effects were reported with semaglutide and discontinuation of treatment due to side effects was reported to be higher than with placebo in one trial (3.4% with semaglutide vs 0% with placebo) [31]. Another trial reported a similar proportion of participants discontinuing treatment due to adverse events (2.4% with semaglutide vs 2.3% with placebo) [33]. 

Semaglutide has been emerging as a promising pharmacotherapy for weight management when combined with lifestyle modifications. Studies have shown it to be significantly more effective for achieving and maintaining weight loss than placebo and liraglutide. The downside is the subcutaneous route of administration which might be inconvenient for some patients, although weekly dosing makes it a bit more convenient than daily subcutaneous dosing of liraglutide. Some patients have not been able to tolerate it. For those who can tolerate semaglutide, it can be added to behavioral therapy and diet modification for achieving and maintaining significant weight loss in the obese/overweight population. The possible superiority of semaglutide with a similar risk of adverse events makes it an excellent choice of drug for the management of obesity in the patient population willing to take subcutaneous medication [22]. 

Table 1 summarizes the relevant clinical studies that evaluated different pharmacological treatments for obesity.

**Table 1:** Summary of Relevant Studies on Pharmacotherapy of Obesity SUCRA: surface under the cumulative ranking curve; CrI: credible interval; MD: mean difference;GLP-1: glucagon-like peptide 1; LDL: low-density lipoprotein

Authors	Number of patients	Randomization	Length of study	Summary of findings
Yu et al., 2013 [14]	67	Three groups, Group 1: Diet alone, Group 2: Diet+orlistat, Group 3: Diet+Orlistat+Exercise	10 weeks	Orlistat in combination with diet and diet plus exercise vs diet alone: decreased body mass index, body weight, waist circumference, and levels of total cholesterol and LDL cholesterol.
Khera et al., 2016 [15]	29,018	Comparison of FDA-approved medications (orlistat, phentermine-topiramate, naltrexone-bupropion, liraglutide) with placebo	NA	23% of those receiving placebo had 5% weight loss compared to 44% of those taking orlistat (OR 2.70, 95% credible interval (CrI) 2.34-3.09. SUCRA 0.22), 75% of those taking phentermine-topiramate (OR 9.22, 95% CrI 6.63-12.85. SUCRA 0.95), 55% of those taking naltrexone-bupropion (OR 3.96, 95% CrI 3.03-5.11. SUCRA 0.60) and 63% of those taking liraglutide (OR 5.54, 95% CrI 4.16-7.78. SUCRA 0.83). After one year, excess weight loss as compared to placebo was 2.6 kg with orlistat (95% CrI -3.04 to -2.16 kg) 8.8 kg with phentermine-topiramate (95% CrI-10.20 to -7.42 kg), 5.0 kg with naltrexone-bupropion (95% CrI -5.94 to -3.96) and 5.3 kg with liraglutide (95% CrI -6.06 to -4.52 kg). naltrexone-bupropion and liraglutide were found to be associated with the highest odds of treatment discontinuation.
Gorgojo-Martínez et al., 2019 [16]	500	Orlistat group VS liraglutide group	7 months	Over seven months, both liraglutide and orlistat led to a reduction of weight (7.7 kg with liraglutide vs 3.3 kg with orlistat), systolic blood pressure, fasting glucose level, LDL cholesterol, and alanine transaminase. 64.7% of participants taking liraglutide lost 5% or more of their baseline weight vs 27.4% with orlistat. Liraglutide also led to lower prediabetes rates compared to orlistat.
Grabarczyk TR 2018 [17]	66,035	Lorcaserin group, phentermine-topiramate group, phentermine group, orlistat group, and MOVE group	6 months	Over 20 weeks phentermine-topiramate group had 4.1% decrease in weight from baseline vs 3.6% in phentermine group vs 2.1% in orlistat group vs 1.6% in MOVE! group (VA’s weight management program) [P < 0.05]. 40.3% of participants taking phentermine-topiramate achieved 5% weight loss from baseline.
Shi et al., 2022 [22]	49,810	NA	NA	Phentermine-topiramate showed MD of percentage body weight change -7.97 (95%CI -9.28 to –6.66), GLP-1 agonists showed MD of -5.76 (95%CI -6.30 to -5,21). Discontinuation of treatment related to adverse events was reported for GLP-1 agonists (OR 2.17, 95% CI 1.71 to 2.77). Of the FDA-approved agents, phentermine-topiramate was the least likely to be discontinued due to side effects. Post-hoc analysis established superiority of semaglutide for weight loss with almost similar risks of adverse effects.
Shibuya et al. ,2019 [23]	3,411	Phentermine hydrochloride, phentermine-topiramate, bupropion-naltrexone, and lorcaserin groups	12 weeks	Average weight loss with anti-obesity medication was 3.45% of body weight from baseline. Participants taking naltrexone-bupropion lost 2.66 ± 5.03% of body weight as compared to 3.63 ± 5.7% with phentermine-topiramate (P < 0.0001)
Lei et al., 2021 [24]	6,740	NA	NA	Phentermine-topiramate was found to be effective in weight reduction as well as waist circumference, blood pressure, lipid level, and plasma glucose level reduction. The efficacy was dose-dependent, and the limiting factor was association of phentermine-topiramate with neurological side-effects
Rubino et al., 2022 [28]	338	Semaglutide group or liraglutide group or placebo group plus diet and physical activity	68 week	Participants taking semaglutide had mean weight change of -15.8% vs -6.4% in those taking liraglutide and -1.9% with pooled placebo (95%CI -12 to -6.8, P < .001). Loss of at least 10% body weight: 70.9% of those taking semaglutide vs 25.6% of those taking liraglutide (OR 6.3, 95%CI 3.5 to 11.2, P < .001)
Astrup et al., 2012 [29]	564	Liraglutide group, orlistat group, placebo group	2 years	Year 1: participants on liraglutide 3.0 mg lost 5.8 kg more weight than placebo (95%CI 3.7 to 8.0) and 3.8 kg more than those taking orlistat (95% CI 1.6-6.0, P 0.0001). Year 2: participants receiving liraglutide 2.4/3.0 mg for whole two years lost 3.0 kg more weight than those receiving orlistat (95%CI 1.3-4.7, P < 0.001). It also showed improvement in lipids and blood pressure along with decreased two-year prevalence of prediabetes by 52% and metabolic syndrome by 59%. Nausea and vomiting were the most frequently reported side effects.
Wadden et al., 2021 [31]	611	Semaglutide plus intensive behavioral therapy vs placebo plus intensive behavioral therapy	68 weeks	Mean body weight change from baseline was -16.0% for semaglutide vs -5.7% for placebo (95% CI -12.0 to -8.6, P < .001). 86.6% lost 5% of body weight with semaglutide vs 47.6% with placebo (P < .001), for 10% body weight: 75.3 % with semaglutide vs 27.0% with placebo (P < .001), for 15% body weight: 55.7% vs 13.2% (P < .001). Gastrointestinal adverse events: 82.8% with semaglutide vs 63.2% with placebo. Discontinuation of treatment due to adverse events: 3.4% with semaglutide, 0% with placebo.
Rubino et al., 2021 [33]	902	Semaglutide plus lifestyle modification vs placebo plus lifestyle modification	68 weeks	From week 20 to 68, mean body weight change with semaglutide was -7.9% vs +6.9% with placebo (95%CI -16.0 to -13.5 P < .001). Improvement was also seen in waist circumference -9.7 cm (95%CI -10.9 to -8.5 cm, P < .001), systolic blood pressure – 3.9 mm Hg (95%CI -5.8 to – 2.0 mm Hg, P < .001) and physical functioning score SF-36 2.5 (95%CI 1.6 to 3.3, P < .001). 49.1% reported gastrointestinal adverse events with semaglutide vs 26.1% with placebo. Discontinuation of treatment due to adverse events: 2.4% with semaglutide vs 2.2% with placebo.

Limitations

This is a narrative review and, therefore, Preferred Reporting Items for Systematic Reviews and Meta-Analyses (PRISMA) guidelines were not fully followed, and a quality assessment of selected studies was not performed. PubMed was the only database used. Insufficient data regarding long-term effectiveness and risks of side effects was available.

Future directions

Further studies need to be conducted, preferably clinical trials, to yield statistically significant results regarding efficacy as well as adverse events and safety of anti-obesity medications. Currently, no clear guidelines for the pharmacotherapy of obesity exist and such studies could help develop them. Moreover, having clear guidelines would likely help ensure insurance coverage of those medications when obesity is the sole indication.

## Conclusions

Medical management should be considered when lifestyle modifications alone are insufficient to maintain weight loss. All five anti-obesity medications approved by FDA, i.e., semaglutide (weekly subcutaneous), liraglutide (daily subcutaneous), phentermine-topiramate (oral), orlistat (oral), and naltrexone-bupropion (oral) appear to be effective but data regarding safety profiles, especially long-term safety, is limited. Semaglutide appears to be the most effective of the five drugs, but tolerability and feasibility of subcutaneous administration remain an issue. Although more research into all of the aforementioned medications is needed, current data provide sufficient evidence to support their use. The pharmacotherapy should be individualized based on co-morbidities, gender and age, and patient preference. Clinicians are still hesitant to prescribe these medications due to concerns about adverse events, but this approach should be reconsidered, as obesity should be treated as a disease with an individualized management plan for the individual patient.
